# The Basis and Advances in Clinical Application of Cytomegalovirus-Specific Cytotoxic T Cell Immunotherapy for Glioblastoma Multiforme

**DOI:** 10.3389/fonc.2022.818447

**Published:** 2022-04-19

**Authors:** Amin Daei Sorkhabi, Aila Sarkesh, Hossein Saeedi, Faroogh Marofi, Mahnaz Ghaebi, Nicola Silvestris, Behzad Baradaran, Oronzo Brunetti

**Affiliations:** ^1^ Student Research Committee, Tabriz University of Medical Sciences, Tabriz, Iran; ^2^ Immunology Research Center, Tabriz University of Medical Sciences, Tabriz, Iran; ^3^ Department of Hematology, Faculty of Medicine, Tabriz University of Medical Sciences, Tabriz, Iran; ^4^ Cancer Gene Therapy Research Center (CGRC), Zanjan University of Medical Sciences, Zanjan, Iran; ^5^ Medical Oncology Unit, Department of Human Pathology "G. Barresi", University of Messina, Messina, Italy; ^6^ Neurosciences Research Center, Tabriz University of Medical Sciences, Tabriz, Iran; ^7^ Medical Oncology Unit-Istituto di Ricovero e Cura a Carattere Scientifico (IRCCS) Istituto Tumori “Giovanni Paolo II” of Bari, Bari, Italy

**Keywords:** immunotherapy, adoptive cellular therapy (ACT), CMV-specific T cell, cytomegalovirus (CMV), herpes virus, glioblastoma multiforme, glioma, brain tumor

## Abstract

A high percentage of malignant gliomas are infected by human cytomegalovirus (HCMV), and the endogenous expression of HCMV genes and their products are found in these tumors. HCMV antigen expression and its implications in gliomagenesis have emerged as a promising target for adoptive cellular immunotherapy (ACT) strategies in glioblastoma multiforme (GB) patients. Since antigen-specific T cells in the tumor microenvironments lack efficient anti-tumor immune response due to the immunosuppressive nature of glioblastoma, CMV-specific ACT relies on *in vitro* expansion of CMV-specific CD8^+^ T cells employing immunodominant HCMV antigens. Given the fact that several hurdles remain to be conquered, recent clinical trials have outlined the feasibility of CMV-specific ACT prior to tumor recurrence with minimal adverse effects and a substantial improvement in median overall survival and progression-free survival. This review discusses the role of HCMV in gliomagenesis, disease prognosis, and recent breakthroughs in harnessing HCMV-induced immunogenicity in the GB tumor microenvironment to develop effective CMV-specific ACT.

## Introduction

Glioblastoma multiforme (GB), also known as WHO‐grade IV glioma, is the most prevalent and lethal primary malignant tumor of the central nervous system (CNS), with a dismal prognosis despite substantial breakthroughs in disease treatment and the introduction of multimodal strategies combining surgery and chemoradiation (1–3). The limited efficacy of current therapeutic schemes is a cause of concern in the therapy of patients. The suboptimal “standard of care” comprises microsurgical tumor debulking, concomitant radiochemotherapy with temozolomide (TMZ), and consolidation TMZ chemotherapy, with a median survival of 15 months and a 5-year survival rate of less than 5%, underlining the imperative need for alternative therapies (4, 5).

Several immunosuppressive mechanisms that characterize the immunological milieu of GB have been investigated in an effort to improve GB treatment strategies. Nevertheless, emerging immunotherapeutic interventions aimed at stimulating specific immune responses targeting solid tumors have provided patients with GB a glimpse of hope over the last decade. At present, cancer immunotherapy may be broadly classified into four categories: i. monoclonal antibodies (mAb) against inhibitory immune checkpoint molecules, ii. oncolytic virotherapy, iii. adoptive cellular therapy (ACT), and iv. cellular vaccine therapy (6). ACT is a highly personalized therapeutic approach that involves a patient’s T cells to deliberately redirect them to induce anti-tumor immune responses, rather than relying on the cytotoxic processes of conventional therapies, over which GB cells are refractory (7). However, the primary issue in this process is to overcome dysfunctional T cells, which are characterized by a loss of effector function, notably impaired cytotoxicity, and reduced secretion of inflammatory cytokines such as interleukin-2 (IL-2), tumor necrosis factor-α (TNF-α), and/or interferon-γ (IFN-γ) (8, 9).

Virus-associated malignancies are viable targets for the ACT, which leverage viral antigens expressed by cancer cells to redirect virus-specific T lymphocytes to eradicate tumor cells. There is a plethora of evidence that human cytomegalovirus (HCMV) antigens are abundantly expressed in GB tissue but not in normal brain tissue and potentially contribute to gliomagenesis (10). However, since the 80-90% prevalence of HCMV in healthy adults does not correspond to the prevalence of GB (1 out of 30,000), HCMV is not labeled as a classic oncogenic virus (11, 12). Thereby, HCMV does not directly induce malignant cellular transformation, but rather contributes to GB pathogenesis *via* “oncomodulation” of host cellular pathways (13). In this context, HCMV is not eliminated from the host body after primary infection, and any potential immunosuppressive condition, notably GB-associated immunodeficiency, reactivates this virus, leading to the activation of oncogenic pathways (14). These insights open up a prospective avenue for stimulating dysfunctional CMV-specific T cells to elicit competent anti-tumor immune responses in order to overcome the heterogeneous and antigen-escaping nature of GB. In this review, we will discuss the implications of HCMV in GB pathogenesis and prognosis, as well as recent advances of CMV-specific T cell therapy in GB patients.

## HCMV Gliomagenesis

**Figure 1:**
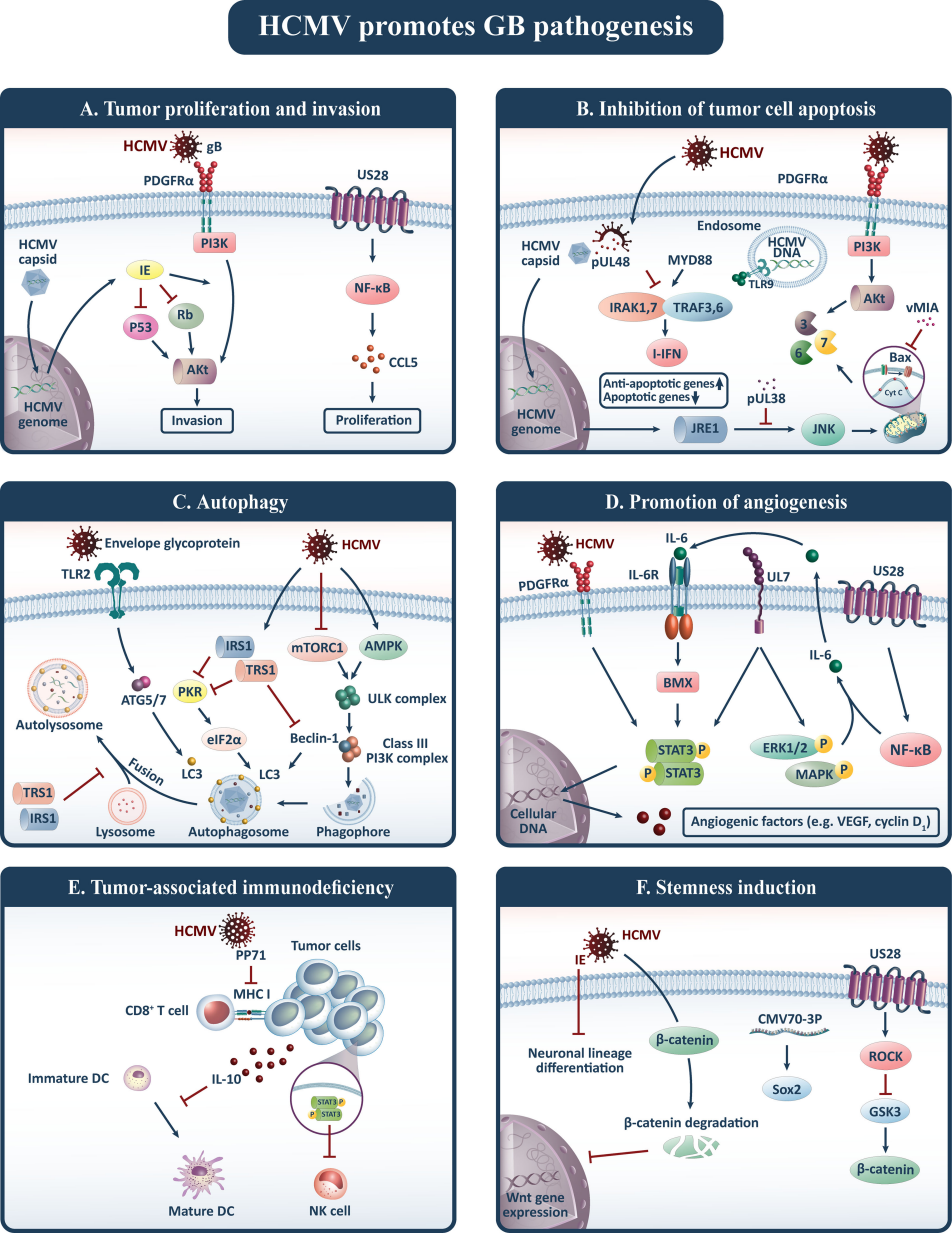
HCMV contributes to gliomagenesis and promotes six oncogenic pathways including: **(A)** tumor proliferation and invasion, **(B)** inhibition of tumor cell apoptosis, **(C)** autophagy, **(D)** promotion of angiogenesis, **(E)** tumor-associated immunodeficiency, and **(F)** stemness induction. Akt, Protein kinase B; CCL5, Chemokine (C-C motif) ligand 5; DC, Dendritic cell; HCMV, Human cytomegalovirus; IE, Immediate-early protein; I-IFN, type I interferon (I-IFN); IL, Interleukin; JNK, c-Jun N-terminal kinase; LC3, Light chain 3; MHC, Major histocompatibility complex; NF-κB, Nuclear factor-kappa B; NK, Natural killer; PDGFRα, Platelet-derived growth factor receptor alpha; PI3K, Phosphatidylinositol-3-kinase; Rb, Retinoblastoma; SOX2, SRY-Box 2; STAT3, Signal transducer and activator of transcription 3; TLRs, Toll-like receptors; TRAF3, TNF receptor-associated factor; Treg, Regulatory T cell; VEGF, Vascular endothelial growth factor; vMIA, viral mitochondria-localized inhibitor of apoptosis.

HCMV has been implicated in gliomagenesis through a variety of distinctive mechanisms, which are classified into six major hallmarks of cancer, including tumor proliferation and invasion, inhibition of tumor cell apoptosis, autophagy, promotion of angiogenesis, tumor-associated immunodeficiency, and stemness induction (15) (**Figure 1**).

### Tumor Proliferation and Invasion

Cell proliferation and invasion in human cancers are among the major areas of concern, leading to uncontrolled disease activity and fatal tumor cell invasion to the surrounding brain tissues, rendering it unresponsive to treatment interventions such as complete surgical resection. Hence explicating the mechanisms associated with tumor cell proliferation and invasion is critical in order to develop effective treatment approaches. HCMV generates oncomodulatory proteins that interact with tumor cell pathways, promoting tumorigenesis and tumor invasion. In this regard, the HCMV immediate-early (IE) proteins, which include IE1-72 or IE2-86, are an essential viral transcriptional activators that have been discovered to be expressed in more than 90% of glioma tumors (16). In particular, the HCMV-IE1-72 protein along with IE2-86 influences human glioblastoma growth by inactivating the p53 and the retinoblastoma (Rb) family of tumor suppressor proteins, resulting in cell cycle progression and apoptosis blockade while also actively engaging in cell cycle arrest to facilitate viral replication (17–20). However, it has been shown that these paradoxical cell cycle transformation mechanisms may enhance cellular proliferation, DNA synthesis, and entry into the cell cycle (21, 22). Furthermore, HCMV glycoprotein B (gB) is the viral abundant envelope glycoprotein that functions in the same way as the genuine ligand, platelet-derived growth factor (PDGF), by binding to the receptor tyrosine kinase (RTK)PDGFR-alpha (PDGFRα) to promote viral cellular entry (23). It has been demonstrated that gB is endogenously overexpressed in GB samples and contributes to the persistent phosphorylation of PDGFRα, protein kinase B (Akt), and Src. As a result, it triggers downstream RTK signaling of an essential oncogenic phosphatidylinositol-3-kinase (PI3K)-Akt axis, which is sufficient to enhance glial precursor proliferation and drive gliomagenesis (24, 25). Another HCMV-encoded protein that contributes to virus latency, cell-to-cell dissemination, immune evasion, and angioproliferative signaling is the chemokine receptor US28, which is extensively expressed in GB samples (26, 27). It has been proposed that US28 activates the hypoxia-inducible factor-1/pyruvate kinase M2 (HIF-1/PKM2) feedforward loop *via* Gα_q_-, CaMKII-, and Akt/mTOR-dependent pathways, directing proliferation, angiogenesis, and metabolic reprogramming in GB cells, and that US28 knockdown reverses proliferation in these cells (28). In agreement with these findings, *in vivo* animal studies established that nanobody-mediated US28 silencing restricts the tumor development of oncogenic glioma cells (29). These findings substantiate the notion that US28 is implicated in HCMV-mediated oncomodulation. Furthermore, immunofluorescence studies suggest that US28 upregulates phosphorylated signal transducer and activator of transcription 3 (p-STAT3) and endothelial nitric oxide synthase (e-NOS) that contribute to invasion of GB cells by upregulating pro-invasive factors including extracellular-signal-regulated kinase 1/2 (ERK1/2), focal adhesion kinase (FAK), and Src (30–32). FAK is an important player of integrin-dependent cell signaling that controls cell adhesion and migration, which are essential for metastasis. US28 constitutively activates FAK *via* phospholipase C- (PLC-), reducing cell adhesion and hence enhancing GB migration (33, 34).

### Inhibition of Tumor Cell Apoptosis

Upon infection, one of the most important underlying mechanisms of viral persistence and replication in cells is the expression of proteins that restrict virus-mediated apoptosis. Studies have shown that HCMV enhances the expression of anti-apoptotic activating transcription factor 5 (ATF5), which is extensively upregulated in glioma cells and contributes to their survival (35). ATF5 tends to bind to an ATF5-specific regulatory element in the B-cell lymphoma/leukemia-2 (Bcl-2) P2 promoter to enhance Bcl-2 expression (36). This enhances anti-apoptotic Bcl-2 expression while decreasing apoptotic Bcl-2-associated X (Bax) protein expression, indicating that HCMV has an anti-apoptotic effect on glioma cells (37). Likewise, HCMV IE86 has been shown to impede apoptotic pathways in glioma cells through promoting heterogeneous nuclear ribonucleoprotein A2/B1 (hnRNP A2/B1)-mediated alternative splicing of Bcl-x, resulting in reduced Bcl-xS/Bcl-xL ratio (38). As well, HCMV-driven Bcl-2 and viral mitochondria-localized inhibitor of apoptosis (vMIA) are endowed with the ability to impede mitochondrial outer membrane permeabilization *via* interaction with Bax and adenine nucleotide translocase, as well as redirection of these proteins to other subcellular locations. As a result, infected cells’ proapoptotic potential is drastically reduced (39, 40). On the other hand, the vMIA interferes with the enzymatic activity of the inner membrane mitochondrial protein ATP synthasome, resulting in impaired phosphate transport and hence decreased mitochondrial ATP synthesis (41). As a consequence, since apoptosis is an energy-requiring process, bioenergy depletion inhibits it.

Furthermore, the HCMV IE1 and IE2 proteins are both mutagenic, intervene with the function of the p53 and Rb tumor suppressors, foster the S-phase, and suppress apoptosis in infected cells (42, 43). In this respect, IE1 expression was shown to significantly downregulate p53 mRNA transcription in a human glioma cell line (44). These viral proteins also hinder apoptosis *via* the PI3K cellular pathway (45). Additionally, HCMV deubiquitinase suppresses the generation of anti-cancer type I interferons (I-IFNs), which contributes to the upregulation of anti-apoptotic genes and down-regulation of apoptosis-inducing genes, encouraging cells to surpass the G1-phase rapidly (46).

### Autophagy

The catabolic program of autophagy is a self-defense mechanism that entails the provision of cellular material for lysosomal degradation to supply energy and macromolecules. This mechanism protects tumor cells from nutrient deprivation, provides the substrates essential for cell survival, and modulates apoptosis (47–49). Evidence suggests that several autophagy-related proteins display a proviral function, implying that HCMV is privileged to utilize components of the autophagic machinery for its advantage. In this context, the cross-talk between autophagy-initiating kinase ULK1 and the HCMV tegument protein pp28 during viral replication has been shown necessary for effective virus release (50). On the other hand, studies on human fibroblasts infected with HCMV have revealed that the virus can induce autophagy by lipidation of microtubule-associated protein 1 light chain 3 (LC3), a hallmark of autophagy (51). In addition, HCMV envelope glycoprotein stimulates toll-like receptor 2 (TLR2) that may contribute to autophagy induction (52).

During the infection of human fibroblasts, HCMV regulates autophagy in two opposed directions. In the early stages of infection, HCMV induces autophagy independent of *de novo* viral protein synthesis, as evidenced by an increment in the number of autophagosomes and autophagic influx, while in the late stages of infection (18-24h post-infection), HCMV suppresses autophagy *via* mechanisms reliant on *de novo* viral protein synthesis (53). TRS1 and IRS1, for example, are two anti-autophagic proteins encoded by HCMV that have been found to act as antagonists for the eukaryotic initiation factor 2α/protein kinase R (eIF2α/PKR) signaling pathway by binding to PKR and thus blocking eIF2α phosphorylation, which has a central role in autophagy induction. However, it has been suggested that the interplay between TRS1 and Beclin 1 is the dominant contributor in this anti-autophagic procedure (53–55).

Overall, HCMV infection necessitates a baseline level of autophagy as well as specific autophagic proteins in order to maintain effective viral morphogenesis. Thus, autophagy in HCMV-infected cells is characterized as an oncomodulator mechanism that enhances tumor cell viability while restricting viral infection in the late stages of infection.

### Promotion of Angiogenesis

HCMV-infected tumor cells may rewire signals to adjacent neoplastic and endothelial cells, perpetuating autocrine and paracrine signaling that fosters tumor cell motility. Endothelial cell invasion and tumor cell neovascularization are involved in the malignant progression of glioma, and the HCMV is a central player in these pathways (56). In support of this notion, studies on a GB mouse model have suggested that murine CMV (MCMV) infection enhances intratumoral blood flow and angiogenesis, which has been linked to platelet-derived growth factor-D (PDGF-D) mediated pericyte recruitment (57). The expression of PDGF-D is further upregulated by nuclear factor-kappa B (NF-κB) signaling, which is mediated by CMV glycoprotein-induced phosphorylation of p56 (58, 59).

Furthermore, research has revealed that HCMV encodes UL7 protein, which serves homology to the N-terminal V-like domain of carcinoembryonic antigen-related cell adhesion molecule 1 (CEA-CAM1), a pro-angiogenic factor involved in signal transduction and cellular adhesion. Experiments on HCMV-infected endothelial cells evidenced that CEA-CAM1phosphorylates STAT3 and ERK1/2 MAP kinases and contributes to the secretion of pro-angiogenic factors such as IL-6. Thus, it provokes angiogenesis under physiological and pathological conditions and enhances the viability of endothelial cells by upregulating the anti-apoptotic factor survivin (60–62). Likewise, HCMV-infected cells are found to express the HCMV-driven US28 protein on their surface, which triggers the NF-κB pathway, directing the production of IL-6 and cyclooxygenase-2 (COX-2). Following that, IL-6 and COX-2 bind to their cognate receptors and induce STAT3-dependent VEGF and Cyclin D1 transcription, hence enhancing angiogenesis (26, 63, 64). Similarly, UL33 is another HCMV-encoded protein linked to an oncogenic signature that overlaps with US28-mediated signaling to some extent. It activates GB angiogenic pathways, including the IL-6-STAT3 axis, VEGF and COX-2 promoters, and hypoxia-induced factor-1 (HIF-1) (65). HIF-1 has been discovered to regulate anterior gradient protein 2 (AGR2) expression, which is involved in the adaptive hypoxia response to stimulate angiogenesis and tumor development in GB (66). Furthermore, HCMV-infected glioma stem cells (GSCs) release CMV IL-10, which interacts with monocytes to enhance CMV transcriptional activity and subverts them to a tumor-supportive M2 macrophage/microglia phenotype. As a result, it initiates a feed-forward loop toward glioma cells by boosting angiogenic VEGF production (30).

Another HCMV-encoded protein that stimulates angiogenesis in GB is phosphoprotein 71 (pp71), which is implicated in the activation of early viral gene expression (67). It has been suggested that pp71 is abundantly expressed in stem-like (CD133+) primary GB cells, especially in high-grade gliomas, and promotes stem cell factor expression *via* an NF-κB-dependent pathway (68, 69). In this way, stem cell factor binds to the c-kit receptor tyrosine kinase, triggering upregulation of endothelial progenitor cell migration and thereby accelerating angiogenesis in hypoxic settings (70). Additionally, the HCMV phosphoprotein65 (pp65) is ubiquitously expressed in 91% of GB tissues and has been observed to colocalize with endocan in the tumor cell cytoplasm (71). Endothelial cell-specific molecule-1 (ESM-1) or endocan is a proteoglycan biomarker of neoangiogenesis whose expression is regulated by VEGF in endothelial cells. The HCMV pp65 protein indirectly enhances endocan expression by modulating VEGF and cytokines such as TNF-α and IL-6, thereby enhancing glioma neovasculature (72).

Importantly, microRNA (miR)-217 and miR-199a-5p expressions are upregulated in HCMV-infected endothelial cells, which can suppress sirtuin 1 (SIRT1) and forkhead box O3 (FOXO3a) endogenously. This culminates in reinforced migration and tube formation of infected endothelial cells (73, 74). Similarly, miR-138 upregulates p-STAT3 protein expression through downregulating SIRT1 expression in HCMV-infected human umbilical vein endothelial cells (HUVECs), stimulating them to migrate and form tubes (75).

Taken together, HCMV promotes angiogenesis through multilateral interactions with stem cells and endothelial cells, resulting in upregulation of angiogenic factors, increased endothelial cell viability, and recruitment to form vessels required to supply tumor cells with adequate amounts of nutrients and oxygen.

### Tumor-Associated Immunodeficiency

Infection with HCMV and its interplay with immune system components disrupt innate and adaptive immune pathways to evade viral elimination, resulting in compromised anti-tumor responses and antigen presentation. HCMV pp71 restrains the accumulation of major histocompatibility complex (MHC) class I in order to regulate protein trafficking and evade virus-specific CD8^+^ T cell recognition (76). Likewise, this viral tegument protein augments C-C motif chemokine ligand (CCL) 2/monocyte chemoattractant protein 1 (MCP1) through transcriptional upregulation, which has been attributed to glioma tumor grade and recurrence (77, 78). Also, HCMV infection tends to increase CCL5 expression, and since this ligand binds to and triggers the US28 pathway, it amplifies US28-induced GB invasiveness (31). On the other hand, US28-mediated recruitment of monocyte and macrophage increases in response to C-X3-C motif chemokine ligand (CX3CL)1/fractalkine elicited by GSC and tumor-associated macrophage (TAM) in the GB niche (34).

Along with monocyte chemotaxis to the tumor niche, HCMV-encoded IL-10, which appears to mimic the immunomodulatory effects of human IL-10, shifts monocyte polarization toward a deactivated pro-tumoral M2c phenotype, limiting CD4^+^ T cell activation and proliferation substantially (79). It hinders MHC class II and the co-stimulatory protein CD86 while raising the co-stimulatory inhibitory molecule B7-H1 (30). Additionally, this viral homolog amplifies its immunosuppressive effects in myeloid cells by upregulating human IL-10 and its positive regulator, tumor progression locus 2 (TPL2), as well as interacting with its receptor. This leads to an increase in heme oxygenase 1 (HO-1) expression, which is associated with immunosuppression (80).

The HCMV-induced immunosuppressive state in GB is exacerbated by additional mechanisms. In this context, research on CMV-infected mouse models has shown that CD4^+^Foxp3^-^IL-10^+^ Tregs contribute to an effective antiviral T cell response and viral clearance, whilst IL-10- and TGF-ß-dependent pathways have been shown to boost anti-inflammatory pathways in GB (81, 82). Thus, Treg-induced TGF-ß-mediated inactivation of the NKG2D receptor might enable tumor cells in GB patients to evade recognition by immune system cytolytic effector cells, resulting in tumor progression (83). Similarly, HCMV-encoded UL18, UL40, and UL142 are viral MHC class I homologs that enhance surface human leukocyte antigen (HLA)-E expression, which binds to the NK cell inhibitory receptor NKG2A/CD94 to resist NK lysis (84–86).

Taken together, HCMV triggers pathways that enable tumor cells to evade immune recognition while also interacting with immune cells, boosting immunosuppressive characteristics and compromising anti-tumor immune responses.

### Stemness Induction

GSCs are neoplastic units with a high potential for unlimited self-renewal, multipotency, and tumor initiation, and they are appropriate hosts for persistent neurotropic HCMV infection as they lack DNA repair mechanisms, facilitate viral immune evasion, and have an extended life span (87). These stem cells endow GB tumors with chemoradioresistance and drive tumor recurrence (88).

Several preclinical studies have shed light on the cross-talk between HCMV infection and stemness induction in GB tumors. HCMV has shown strong tropism to GSCs with high expression of CD133 (30). Similarly, studies have shown that GSCs infected with HCMV outlive and contain greater amounts of viral DNA compared to glioma cell lines, which coincides with upregulation of stemness markers in GSCs (89). Of note, the stemness markers are expressed in GB tumors with interdependence on HCMV IE expression, rendering tumor-derived GSCs unable to differentiate into astrocytic or neuronal phenotypes, promoting GSCs features and therefore increasing tumor aggressiveness (90). It was suggested that HCMV IE1/IE2 is co-expressed with stemness markers including CD133, Nestin, and SRY-box 2 (Sox2) and is implicated in the miR-45-Sox2 axis, resulting in enhanced self-renewal of GSCs. Accordingly, IE1/IE2 knockdown by an RNA-i-based strategy limits Sox2 expression, tumorsphere formation, and induces apoptosis in HCMV positive GSCs (43). These findings provide credence to the notion that HCMV IE protein expression has a significant detrimental predictive role in GB tumors through induction of stemness properties (91).

Moreover, the HCMV-encoded CMV70-3P miRNA is expressed in GB tissues and upregulates Sox2 expression, which is linked to stemness induction. In this respect, studies have demonstrated that suppression of CMV70-3P impedes tumor migration and invasion, indicating that it is a promising target for GB therapy (92).

## Expression of HCMV in GB

Beyond the proposed oncomodulatory mechanisms of HCMV implicated in gliomagenesis, the detection of HCMV in GB tissue has emerged as a debate with many incongruous and contradictory results since 2002. These are primarily attributed to methodological discrepancies; yet, these findings offer a viable path for translating these viral protein expressions into promising anti-HCMV therapies to approach GB (10). To this end, several studies have employed immunohistochemistry and *in situ* hybridization techniques to detect HCMV, which are capable of illuminating the site of virus particles and RNA accumulated within cancer tissues but are predisposed to user errors. Others have employed a sensitive nested polymerase chain reaction (PCR) approach that is sensitive enough to detect extremely low concentrations of viral DNA utilizing robust amplifying strategies (93–95). Despite this, recent PCR assays failed to identify CMV pp65, gB, and IE in GB patients’ peripheral blood or tumor samples (93, 96, 97). Other investigations, on the other hand, highlighted the significance of time in the test’s sensitivity. Herpesviral genomes inside cells become resistant to PCR amplification over time as a consequence of physical deterioration, reflecting that the most recent samples can be detected to express HCMV genes much more abundantly (98, 99). However, HCMV nucleic acid and immunodominant proteins such as pp65 and IE1-72 have been detected in a significant proportion of GB samples through *in situ* hybridization techniques (10, 16, 100–102). Others, using sensitive PCR, immunohistochemistry, and *in situ* hybridization, discovered that a significant proportion of GB samples contained HCMV antigen and DNA and that the expression rate may vary depending on glioma grade, with high-grade gliomas expressing viral antigens more than low-grade gliomas (95, 100, 103). In another study, researchers employed all three techniques to analyze HCMV expression in high-grade gliomas both retrospectively and prospectively, and though they found no significant expression of HCMV regardless of the approach except in low levels in three out of 18 plasma samples at baseline and only one in follow-up (104). In line with these studies, others in various investigations attempted alternative approaches like droplet digital PCR (ddPCR) and next-generation sequencing (NGS) technologies but were still unable to detect HCMV in GB samples (105, 106). The studies also provided insight on the possibility of false-positive results attributable to unidentified cross-reactivity among HCMV antibodies that bind to non-viral human proteins, as well as how varying concentrations of HCMV monoclonal antibodies might contribute to false-positive staining (104, 107). Taking all of the controversies into account, a recent systematic review of 81 studies recruiting 7024 GB samples and 2420 blood samples revealed that HCMV is expressed in 36% and 45.2% of samples, respectively (108). Importantly, none of the healthy surrounding brain tissues employed as a control counterpart were detected to express HCMV genes or their products, indicating a high level of protection against off-tumor cytotoxicity for potential CMV-based immunotherapeutics for GB patients (72, 109–112).

Although the detection of HCMV in GB samples has remained controversial, the inevitability of HCMV oncomodulatory pathways in gliomagenesis has raised the topic of the implications of HCMV infection for disease prognosis. A case-control study revealed that low-grade HCMV infection in GB patients is linked to prolonged survival, and HCMV infection was determined to be a negative prognostic indicator in GB patients (113). Further, the same author conducted a retrospective study and indicated that HCMV infection is a predictive marker and that the low-grade infection coincided with a median 20-month longer survival compared to high-grade infected GB patients (91). These findings, however, appear to be achieved in the exclusion of considering additional prognosticators, such as isocitrate dehydrogenase 1 (IDH-1) mutational status, O6-methylguanine methyltransferase (MGMT) methylation status, TMZ medication status, and so on. On the contrary, the majority of evaluations found no predictive significance for HCMV infection in GB patients (110, 114–116). Nonetheless, a recent meta-analysis of 7 studies and around 500 patients found no statistically significant impact of HCMV infection on GB prognosis (117).

## CMV-Specific Adoptive T Cell Therapy

The expression of HCMV antigens in GB tissues but not in healthy brain tissues primes an intriguing avenue to exploit viral antigens to extend pre-existing antiviral immunity for GB therapy. It has led to a rigorous clinical evaluation of the safety and prospective therapeutic value of autologous CMV-specific T cell administration as a consolidative therapy for recurrent GB. To that aim, researchers employ *in vitro* stimulation to redirect polyfunctional CMV-specific CD8^+^ T cells to target immunodominant HCMV antigens and elicit an effective anti-tumor immune response (118).

### Characteristics of CMV-Specific T Cells Derived From GB Patients

In healthy individuals, CMV-specific CD8^+^ and CD4^+^ T cells account for 10% and 9% of blood CD8^+^ and CD4^+^ T cells, respectively (119). Aside from the intact large T-cell response, the phenotype of CMV-specific T cells appears to be distinctive, as evidenced by their terminal differentiation state. In terminally differentiated CMV-specific CD8^+^ T cells, there is a high expression of NK receptor CD57, which is associated with immunosenescence, as well as cytokines IFNγ and TNF-α, and the cytotoxic molecules granzyme B and perforin. Additionally, these cells lack expression of co-stimulatory CD27 and CD28 molecules that contribute to expansion and activation of T cell receptor (TCR)-stimulated T cells (120).

Preoperative and postoperative flow cytometry analysis of blood from CMV-seropositive GB patients showed that CD4^+^CD57^+^ and CD4^+^CD28^-^ T cells were more abundant than their control counterparts, which were found to have a negative impact on survival in these patients (121). Since the CD4^+^CD28^-^ phenotype is specific to HCMV infection, it is postulated that HCMV antigens expressed in GB tissues persistently stimulate TCRs and drive the expansion of CD4^+^ T cells that express CD57 and subsequently lose the activation marker CD28, indicating defect proliferative capacity and senescence of these cells (121, 122). However, further analysis revealed that the precursor frequency of CMV-specific CD8^+^ T cells was within the range typically observed in healthy virus carriers, and a significant fraction of these cells were positive for the CD57 with CD27^-^CD57^+^ CMV-specific T cells being more frequent in GB patients compared to healthy virus carriers (123).

The CMV-specific T cells lack optimal cytotoxicity in the GB tumor microenvironment due to hypofunctionality in the production of multiple cytokines. In this respect, investigations have shown that exposure of GB patients’ peripheral blood to CMV-pp65 antigen does not lead to a substantial rise in IFNγ production (124). In the context of hypo/dysfunctionality of CMV-specific T cells in GB patients, it was suggested that the large proportion of CMV-specific T cells (60-70%) had attenuated polyfunctionality in expressing TNF-α, IFNγ, macrophage inflammatory protein-1β (MIP-1β), or CD107a (a marker for CD8^+^T-cell degranulation), which may be reconstituted to some extent with *in vitro* stimulation (123). Furthermore, CMV-specific CD8^+^ T cells isolated from resected GB tumor tissue disclosed the same disabilities in expressing IFNγ, TNF, IL-2, or CD107a and lacking expression of the marker for tissue-resident T cells, CD103. Likewise, tumor-infiltrating CMV-specific T cells express augmented levels of inhibitory receptors programmed cell death protein 1 (PD-1), T cell immunoglobulin and mucin-domain containing molecule-3 (TIM-3), and cytotoxic T-lymphocyte-associated antigen 4 (CTLA-4) and lower levels of transcription factors T-bet, eomesodermin (EOMES), and lymphoid enhancer-binding factor 1 (LEF-1), while having a 4-fold lower frequency in tumor tissue compared to T cells circulating in peripheral blood (125). The overexpression of inhibitory receptors in CMV-specific T cells is potentially the basis for the inefficiency of these cells to respond to persistent antigen exposure, which is commonly referred to as immunological exhaustion (126).

### CMV-Specific T Cell Manufacture for ACT

**Figure 2:**
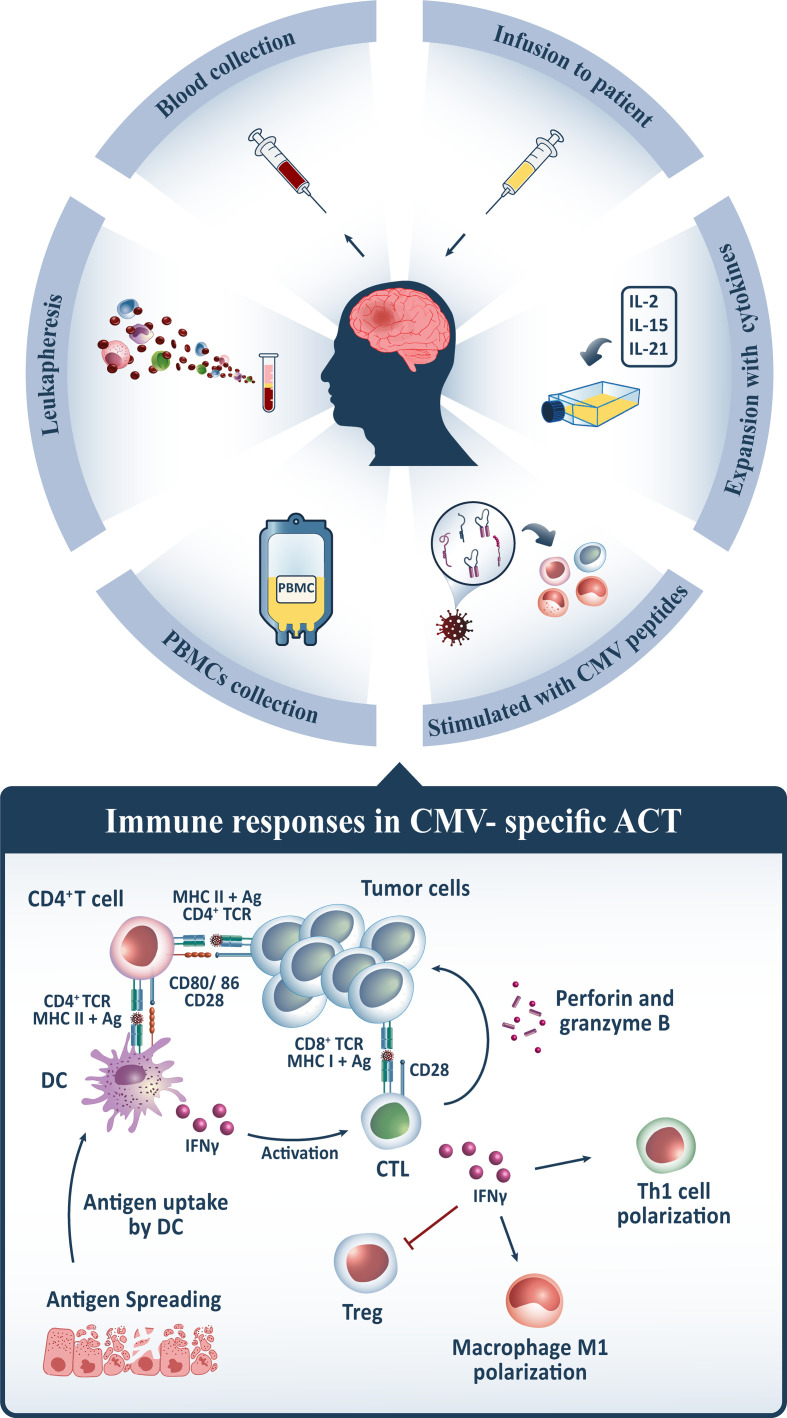
For CMV-specific ACT, PBMCs from GB patients’ peripheral blood are harvested, stimulated with CMV peptides, expanded *in vitro* by conditioning with multiple cytokines such as IL-2/IL-15/IL-21, and afterward reinfused intravenously. Following administration, CMV-specific CD8^+^ T cells traffic to tumor sites and recognize CMV antigens expressed on GB tumor cells, whilst interactions between CMV-specific CD4^+^ T cells and dendritic cells (DC) maintain its activation *via* IFN-γ release. CD8^+^ T cells release granzymes and perforin, which contribute to tumor cytolysis, and IFN-γ, which indirectly provokes other immune cells to elicit the desired anti-tumor immune response. It induces tumoricidal M1 polarization in macrophages, T helper1 (Th1) polarization, and inhibits regulatory T cells (Tregs) in the tumor microenvironment, which is associated with immunosuppression. Upon tumor cytolysis, tumor antigens are released, which are taken up by DCs and presented to cytotoxic T lymphocytes (CTLs), activating them and triggering a multi-antigen directed anti-tumor response.

To begin the process of manufacturing polyfunctional CMV-specific T cells for the ACT, peripheral blood mononuclear cells (PBMCs) are harvested from peripheral blood through leukapheresis, resuspended in the growth medium, and subsequently stimulated with specified HLA class I and II-restricted peptide epitopes from HCMV antigens, including pp65. However, other approaches comprising CMV-pp65 alone, CMV-pp65 coupled with tumor-associated antigens (TAA), or even no antigen are applicable in this procedure. Thereafter, the peptide-pulsed cells are incubated and supplied with recombinant cytokines, including IL-2, which is introduced on day 0 and continued every 2 to 3 days. After 14 days of expansion, the sterility and microbiological tests, as well as the intracellular cytokine assays, are undertaken to qualify them for cryopreservation until future use. For adoptive transfer, T cells are thawed into clinical-grade normal saline and reinfused intravenously (123, 125, 127–129) (**Figure 2**).

Although the proposed procedure varies across trials, the key objective is to reconstitute polyfunctional CMV-specific T cells with adequate quantity, functional quality, high affinity, maturation, differentiation, and homing potential to elicit the desired clinical response in GB patients. In this respect, Luo et al. suggested an alternate cytokine cocktail consisting of IL-2, IL-15, and IL-21 to promote the expansion of high-affinity CMV-specific T cells with a homing phenotype (CCR6^+^ CXCR3^+^) and Th1 polarization (130). To elaborate, IL-2 is a commonly employed cytokine for T cell expansion that has concentration-dependent effects with some counter-productive consequences in the ACT setting (131, 132). IL-2 favors activation-induced cell death (AICD) and stimulates a variety of short-lived effector and long-lived memory T cell responses; however, it also contributes to the development, maintenance, and activity of Tregs, particularly the immunosuppressive phenotype, CD4^+^CD25^+^FOXP3^+^, which limits effector T cell response and proliferation (133, 134). To compensate for the negative impacts, the addition of IL-15 inhibits AICD and contributes to the development and persistence of long-lived memory CD8^+^ T cells. While the addition of IL-21 suppresses Tregs expansion and cooperates synergistically with IL-15 to boost antigen-specific CD8^+^ T cells expansion and IFNγ production, and both, unlike IL-2, induce suitable early-differentiated antigen-specific CD8^+^ T cells for immunotherapeutic applications (135–141). As well, the critical challenge in manufacturing methods is the migratory features and competency of CMV-specific T cells trafficking to the target region. Since healthy virus-naive individuals’ CMV-specific CD8^+^ T cells lack expression of CCR6, CXCR3, and CCR4, enhanced expression of CCR6 and CXCR3 *via* IL-2/IL-15/IL-21 conditioning might be a feasible approach to enhance CMV-specific CD8^+^ T cell trafficking to infection sites and eliciting anti-tumor secretions such as IFNγ and TNF-α (130).

### Proposed Mechanism of CMV-Specific ACT

The purpose of contemporary immunotherapy is to enhance the function of cytotoxic T lymphocytes (CTLs) within the tumor microenvironment, boost CTL priming, and develop a long-term and effective anti-tumor immune response. To that aim, it is anticipated that after administration of CMV-specific T cells, these cells will traffic to tumor sites and interact with CMV-positive tumor cells to trigger cytolysis, therefore limiting tumor proliferation and progression. However, there are certain CMV-negative cells in the tumor tissue that may be targeted to some extent by ACT products. CD8^+^ T cells are a subset of lymphocytes that are specialized to recognize antigenic peptides presented by MHC class I molecules expressed by all tumor cell types; however, since persistent antigen exposure contributes to irreversible commitment to immunological exhaustion, CD4^+^ T cells are required to maintain the CD8^+^ T cell response and prevent exhaustion (142–145). CD4^+^ T cells interact with antigens and enable DCs to optimize antigen presentation and to deliver specific cytokines and co-stimulatory signals to encourage clonal expansion and differentiation of CD8^+^ T cells into an effector or memory T cell (146). Further, some DCs efficiently induce antigen cross-presentation of exogenous antigens on MHC class I molecules to activate CD8^+^ T cells through secretion of IFNγ (147). Following *in vitro* stimulation with immunodominant peptides, the CMV-specific CD8^+^ T cells reverse their competent cytolytic function to release several cytokines, including TNF-α and IFNγ that are involved in several anti-tumor immune responses. Upon antigen recognition, CTLs target tumor cells either directly through triggering synaptic exocytosis of cytotoxic granules comprising perforin and granzymes or indirectly through directing other immune components of tumor microenvironments toward tumor cells *via* secretion of cytokines, including IFNγ and TNF-α (148). IFNγ stimulates tumoricidal M1 macrophage polarization while also upregulating antigen-presenting machinery components such as MHC class I and II, and its synthesis and activities are amplified further by a positive feedback loop arising from inducing Th1 polarization. As well, increased IFNγ secretion suppresses Tregs in the tumor microenvironment (149). Of note, the potential role of CMV-specific ACT on T cell responses to other TAAs expressed in GB has been demonstrated (118). Following tumor cytolysis, CMV-specific ACT might have a bystander effect that extends T cell responses *via* epitope spreading. The antigen-presenting cells, including macrophages and DCs, engulf released antigens and cross-present them to T cells in order to prime CTLs, culminating in a multi-antigen directed immune response that targets a wide range of tumor cells (150) (**Figure 2**).

### Preclinical and Clinical Findings of CMV-Specific ACT

In certain clinical settings, ACT employing genetically modified or unmodified antigen-specific T cells has resulted in long-term and promising clinical outcomes (151). Recent clinical trials have outlined the significance of CMV-specific ACT preceding GB tumor recurrence in being low-risk and offering enhanced progression-free survival and overall survival with compelling effective CMV-specific T cell response (**Table 1**).

**Table 1:** Summary of clinical evidence on CMV-specific ACT.

Tumor	Sample size	Intervention	Results	Reference
Recurrent GB	1	4 IV infusions of 4×10^6^CMV-specific CTLs, 14-28 days apart + TMZ every 28 days for 5 days	Following *in vitro* stimulation, the rate of polyfunctional CMV-specific CTLs was greater than 30%. MRI scans confirmed considerable improvement in appearance one month following the final fusion, and clinically stable condition continued for 17 months.	(123)
Recurrent GB	13	3 to 4 IV infusions of 25 to 40×10^6^CMV-specific CTLs, 14 days apart	CMV-specific ACT in combination with chemotherapy was well tolerated, with minimal side effects, of which none were severe. The median overall survival since the first recurrence was 403 days, with 4 out of 10 patients completing therapy progression-free. These therapeutic approaches have been shown to be safe and to induce long-term clinical stability.	(125)
HER2^+^ recurrent GB	17	A single or more IV infusion of 1×10^6^/m^2^ -1×10^8^/m^2^ HER2 CAR CMV pp65-bispecific CTLs without prior lymphodepletion	Seven of the 16 evaluable patients had stable disease for 8 weeks to 29 months, one patient had a significant reduction in tumor volume that lasted for more than 9 months, and 8 patients progressed. The median survival time from diagnosis and ACT was 24.5 and 11.1 months, respectively. Further, patients who didn’t receive alvage therapy prior to ACT exhibited significantly higher median overall survival than those who received salvage therapy prior to ACT (27.2 months VS 6.7 months)	(152)
Primary GB	22	Arm I (n=8): A single intradermal infusion of 3 × 10^7^/Kg CMV pp65-specific CTLs with 2 ×10^7^ pp65-DCs (CMV-ATCT-DC) + 2 intradermal vaccines with 2 ×10^7^ pp65-DCs	In contrast to the control arm, patients who received CMV-ATCT-DC had significantly higher overall frequencies of IFNγ^+^, TNF-α^+^, and CCL3^+^ polyfunctional, CMV-specific CD8^+^ CTLs.	(153)
		Arm II (n=7): A single intradermal infusion of 3 × 10^7^/Kg CMV pp65-specific CTLs with saline (CMV-ATCT-Saline) + 2 intradermal infusions of saline		
Recurrent and primary GB	65	Arm I (recurrent GB): TMZ PO QD, on days 1-21 + 1 to 4 IV infusions of 5×10^6^-10^8^CMV-specific CTLs, on day 22 + Surgery, on day 30	Despite a 26% failure rate in T cell expansions, repeated infusions led to an increase in CMV-specific CTLs with no dose-limiting toxicities. The median progression-free survival and overall survival were 1.3 and 12 months, respectively. The clinical responsiveness was found to be significantly confounded by MGMT methylation status.	(129)
		(Repeated up to 4 cycles every 42 days and continued with TMZ PO QD, on days 1-21, up to 12 cycles every 42 days)		
		Arm II (primary GB): TMZ PO QD, on days 1-21 + 1 to 4 IV infusions of 1×10^8^ CMV-specific CTLs, on day 22		
		(Repeated up to 4 cycles every 42 days and continued with TMZ PO QD, on days 1-5, up to 12 cycles every 28 days)		
Primary GB	28	Up to 6 IV infusions of 2 × 10^7^CMV-specific CTLs/m^2^ body surface area every 2-4 weeks	CMV-specific ACT was shown to elicit a bystander effect on nonviral tumor-associated antigens *via* antigen spreading.	(118)
			It was discovered that commencing ACT before recurrence had a significantly better influence on median overall survival than commencing it after recurrence (23 months VS 14 months). Overall survival was found to be impacted by many variations in T cell transcriptional patterns at the gene and pathway levels.	
			There was no indication of toxicity associated with CMV-specific ACT.	

ACT, Adoptive cell therapy; CL3, Chemokine (C-C motif) ligand 3; CTL, Cytotoxic T-Lymphocyte; MGMT, O6-methylguanine methyltransferase; TMZ, Temozolomide; IV, intravenous; CMV, cytomegalovirus; DC, dendritic cell; PO, per oral; QD, once per day; IFNγ, interferon-γ; TNF-α, tumor necrosis factor-α; CCL3, CC chemokine ligand 3.

Ghazi et al. discovered that in the peripheral blood of CMV-seropositive GB patients, the frequency of CMV-specific T cells, particularly those specific for the immunodominant pp65 antigen, was considerably lower than in healthy controls, implying the viability of polyclonal CMV-specific T cell generation *via* antigen-presenting cells (APCs) transduced with an adenoviral vector encoding IE1 and pp65 to kill CMV-positive target tissue (127). Further, Crough et al. administered four intravenous infusions of 40×10^6^ CMV-specific CD8^+^ T cells to TMZ-treated patients at 14-28-day intervals in order to evaluate the polyfunctionality of these cells *in vivo*. It was found that *in vitro* stimulation of autologous CMV-specific CD8^+^ T cells induces a more than 20% rise in the proportion of polyfunctional virus-specific T cells, as well as a decrease in CD57^+^ antigen-specific T cells and an increase in CD27^+^ antigen-specific T cells. These led to a substantial decrease in the degree of enhancing tissue as evidenced by magnetic resonance imaging (MRI) scans and a marked improvement in other clinical parameters such as motor-neuron function (123).

For the first time in 2014, Schuessler et al. completed a phase 1 clinical trial in which they administered 3 to 4 infusions of 25 to 40×10^6^ autologous CMV-specific T cells to 13 patients with recurrent GB, yielding the following findings: i, the clinical outcomes in these patients were found to be dose-independent of CMV-specific T cell infusion; ii, the clinical outcomes were unaffected by the detrimental consequences of concurrent standard therapeutic approaches; iii, CMV-specific ACT enhanced the median overall survival (>57 weeks), median progression-free survival (>35 weeks), and progression-free rate by 40% since the first recurrence, with only mild to moderate complications such as headaches or fatigue, none of which were serious (125). Conversely, Weathers et al. suggested that CMV-specific T cells, unlike CMV-negative T cells, exhibit anti-GB activity; despite the fact that seropositivity to CMV does not ensure tumor sensitivity to CMV-specific T cells, and failure in *ex vivo* expansion of CMV-specific T cells is highly attributable to the prior application of TMZ and other cytotoxic chemotherapies. Besides, they found that in spite of favorable enhancements in expressions of CD107a, TNF-α, IFNγ, IL-2, or effector memory markers (CD3^+^/CD8^+^/CCR7^-^/CD45RA^-^) during *ex vivo* expansions, there were no considerable alterations upon adoptive CMV-specific T cell transfer (129). Likewise, the stage of the disease impacts clinical responsiveness to ACT. In this regard, Smith et al. found that initiating adoptive CMV-specific T cell transfers prior to tumor recurrence, rather than thereafter, is associated with a 2.5 and 1.6-fold higher median progression-free survival (10 months vs 4 months) and median overall survival (23 months vs 14 months) since diagnosis, respectively (118).

Moreover, Nair et al. suggested in a preclinical study that autologous T cells stimulated with autologous DCs that were pulsed with CMV pp65 RNA could expand CMV pp65-specific T cells up to 10- to 20-fold, with a substantial rise in IFNγ^+^/CD4^+^ and IFNγ^+^/CD8^+^ cells (154). In line with these findings, Reap et al. performed a randomized clinical trial to assess the efficacy of combining DCs pulsed with CMV pp65 RNA with CMV pp65-specific T cells. They discovered that a single intravenous dose of 3×10^7^ CMV pp65-specific T cells/kg in conjunction with three separate intradermal infusions of 2×10^7^ DCs, as compared to a control group, had a significant influence on the frequency of polyfunctional CMV pp65-specific CD8^+^ T cells with enhanced capacity of simultaneous IFNγ, TNF-α, and CCL3 expression (153).

Since the heterogeneity of CMV pp65 antigen expression might be a major constraint in this therapeutic approach, some clinical trials have used genetically engineered CMV-specific T cells that express chimeric antigen receptors (CARs) to target GB-related surface proteins. Since GB tissues exhibit ineffective antigen presentation for T cell activation and express inhibitory surface ligands including PD-L1 and CTLA-4, the CAR construct incorporates co-stimulatory domains and the extracellular ligand-binding domain to bind the immunosuppressive signal and therefore decouple this binding from any immunosuppressive effects (155–157). To that aim, Ahmed et al. conducted a phase 1 clinical trial where they infused 1×10^6^/m2-1×10^8^/m^2^ pp65-specific T cells grafted with a second-generation human epidermal growth factor receptor 2 (HER2) CAR with a CD28.zeta signaling domain to 17 CMV seropositive patients with HER2^+^ recurrent GB who hadn’t undergone prior lymphodepletion. It resulted in partial clinical response and long-term disease stability in 1and 7 patients, respectively, as well as an increase in median overall survival (27 months since the first infusion). These findings pave the way for the development of CAR CMV bispecific immunotherapy in GB patients (152). However, further large-scale trials are warranted to develop an optimal protocol for incorporating adoptive transfer of CMV-specific T cells with appropriate genetic modifications, as well as DC vaccinations, to approach the GB patients’ treatment.

### Influential Factors in Clinical Responsiveness to CMV-Specific ACT

Several cellular compositions and molecular characteristics of the tumor tissue have been established as having a substantial impact on clinical response to CMV-specific ACT in GB patients. As an instance, excessive PD-L1 expression in GB tissues has been identified as a significant pre-therapy marker that plays an essential part in ACT failure. According to Walker et al., long-term follow-up of patients who received CMV-specific ACT demonstrated that the frequency of PD-L1^+^ cells in tumor tissue prior to therapy correlated adversely with patient survival (158). It has been shown that the PD-1/PD-L1 axis contributes to CTL exhaustion and Treg augmentation, which protects tumor cells from CTL-mediated lysis (159). These findings call for more research into integrating anti-PD-L1 or genetically engineered T cells with PD-1 receptor blockade with CMV-specific ACT to maximize the clinical response of GB patients with predominant PD-L1 expression (160).

Furthermore, despite a paucity of evidence to indicate statistical relevance, additional cellular compositions such as CD3 and Sox2 have been linked to clinicopathological characteristics (158). Sox2 is a stemness marker involved in a variety of cellular activities, associated with maintaining the embryonic and pluripotent stem cell properties. It has been found to be overexpressed in high-grade gliomas, where it is linked to GSC generation and, therefore, poor prognosis (161–163). Sox2 expression is inversely correlated with CD3^+^ T cell infiltration, and patients with long-term survival had a relatively greater number of tumor-infiltrating CD3^+^ T cells (164).

MGMT, a cellular DNA repair enzyme that neutralizes the cytotoxic effects of alkylating drugs like TMZ, is another important biological component related to clinical response in GB patients (165). It has been demonstrated that MGMT promoter methylation is a mechanism of MGMT regulation in gliomas, which is a favorable predictor of progression-free survival and overall survival in TMZ-treated patients (166). In this respect, Weathers et al. revealed that GB patients with MGMT promoter-methylated tumors responded better to a combination of lymphodepleting dose-dense TMZ and CMV-specific ACT and that MGMT methylation status was a determining factor in radiographic outcomes (129). TMZ-induced lymphodepletion prior to ACT commencement exerts multilateral impact on the efficacy of ACT. Eliminating lymphocytes in the tumor microenvironment facilitates infused cells’ trafficking to the tumor site and their accessibility to various homeostatic cytokines, including IL-2, IL-7, and IL-15, while also reducing immunosuppressive cells that impair antigen-presentation. However, these outcomes are transient and will necessitate compensatory actions (167). Conversely, if immunotherapy is delivered shortly after or even during TMZ treatment, the immunosuppressive consequences will restrict immune cells from eliciting a robust anti-tumor response, signifying the timing of TMZ application for these patients. Nonetheless, large-scale immunological surveillance and clinical trials are warranted in order to optimize TMZ combination with CMV-based immunotherapeutic strategies (168).

Aside from MGMT promoter methylation and TMZ treatment, Walker et al. suggested that the presence of an IDH mutation is associated with a considerably better overall survival in GB patients (158). It was discovered that in IDH-mutated GB tumors, PD-L1 expression is downregulated relative to its IDH-wild-type counterpart, which correlated with greater T cell activation (169–171). On the other hand, IDH mutations were speculated to have immunomodulatory effects on both the natural course of disease and response to therapy, since IDH-mutated GB tumors exhibit low infiltration of TAMs, neutrophils, and myeloid cells, which is consistent with a survival benefit (172).

Moreover, Schuessler et al. and Smith et al. established that a signature of several genes in ACT products determines prolonged progression-free survival in patients with recurrent GB, including genes associated with T cell transcription factors, cytotoxic factors, checkpoint markers, and homing markers, as summarized in **Table 2**. The expression profile of these genes in ACT products contributes to CTL proliferation, cytotoxicity, chemotaxis, and interactions with multiple immunosuppressive and oncogenic pathways associated with tumor progression (118, 125).

**Table 2:** The genetic profile of ACT products and their association with antitumor immune response.

	Gene	ACT’s preferred expression	Function	Reference
*T cell transcription factors*	EOMES	High	Full effector development of anti-tumor CTLs	(173)
	BCL6	High	Memory T cell differentiation CD8^+^ T cells proliferation Enhancement of IFNγ production in CD8^+^ T cells	(174, 175)
	FOXP3	Low	Immunosuppressive Treg-specific biomarker HO-1 upregulation Impairment of T cell proliferation	(176, 177)
*Cytotoxic factors*	IFNG	High	Inhibition of glioma growth Restriction of glioma neovascularization Induction of apoptosis Promotion of tumor immunogenicity	(178)
	CST7	Low	Inhibition of granzymes activators including the major pro-granzyme convertases, cathepsins C and H Impairment of T cell cytotoxicity	(179)
	KLRD1/CD94	Low	Immunosuppression through upregulation of TGF-β	(180)
	KLRG1	Low	Impairment of T cell proliferation Impairment of effector cytokines production Immunosuppression Enhancement of proinflammatory cytokines production	(181, 182)
	GZMH	High	Induction of apoptosis	(183)
	PTPN6	Low	Negative regulation of TCR signaling	(184)
*Checkpoint markers*	CTLA-4	Low	Negative regulation of T cell activation Disruption of the co-stimulatory signaling and function	(185)
	XAF1	High	Positive regulation of IFN-induced apoptosis Promotion of p53-mediated apoptosis Promotion of caspase-mediated apoptosis	(186, 187)
*Homing markers*	CCL5	Low	Promotion of glioma growth Enhancement of cancer motility Attraction of anti-inflammatory, pro-tumor effector cells	(188, 189)
	ITGAL/CD11a	High	Initiation of immunological synapse between CTL and tumor	(190)(191)(192)(193)

ACT, Adoptive cellular therapy; BCL6, B-cell lymphoma 6 protein; CCL5, Chemokine (C-C motif) ligand 5; CTLA-4, Cytotoxic T-lymphocyte-associated protein 4; CYST7, Cystatin-F; EOMES, Eomesodermin; FOXP3, Forkhead box P3; GZMB, Granzyme B; IFNG, Interferon gamma; ITGAL, Integrin Subunit Alpha L; KLRD, Killer Cell Lectin Like Receptor D1; PTPN6, Protein tyrosine phosphatase non-receptor type 6; XAF, XIAP Associated Factor 1.

Taken together, there is still a significant necessity to elucidate key factors in clinical responsiveness to CMV-specific ACT, which is acknowledged as an essential milestone toward developing more effective therapeutic approaches and eliminating the obstacles that contribute to treatment failure.

## Conclusion

The most demanding aspect of developing immunotherapeutic approaches for solid tumors is identifying ideal tumor-specific antigens that can be targeted by immunotherapy while avoiding off-tumor toxicities and adverse effects. Like many difficult to treat solid cancers, GB tumors lack expression of antigenic epitopes and induce immunological exhaustion, both of which contribute to the tumor’s low immunogenicity and failure in immunotherapies. Hence, even minor evidence of tumor-specific antigen represents a ray of light for the development of effective antigen-directed immunotherapies. In this way, despite its controversial aspect, the concept of HCMV genes and their products expressions in GB tissues has attracted the interest of researchers seeking to translate it into an effective immunotherapeutic method for GB therapy. Particularly, studies have emphasized the exclusive expression of these antigens in GB tissues rather than adjacent healthy non-tumor tissues, indicating that CMV-specific immunotherapies outperform other methods owing to the lack of off-tumor CNS toxicities in this strategy. Several strategies, including antivirals, CMV antigen-pulsed DC vaccination, and CMV-specific ACT, have been evaluated in clinical trials, with encouraging indications of improved overall survival and progression-free survival in GB patients with minimal adverse effects.

In this review, we discussed HCMV implications for gliomagenesis and outlined various oncogenic pathways that HCMV modulates. Afterward, we discussed various studies with contradictory findings that have established HCMV expression in gliomas and its linkage with disease prognosis. Finally, the features of pre-existing anti-HCMV immunity were described, as well as their stimulation to efficiently target GB tumors. In summary, CMV-specific lymphocytes in the tumor microenvironment exhibit evidence of immunological exhaustion and senescence, making them unable to elicit the desired anti-tumor response, due in part to their specific nature to terminally differentiate and in part to the immunosuppressive nature of GB. As a result, employing immunodominant antigens of HCMV and *ex vivo* pre-conditioning with cytokines to reconstitute their cytotoxic activity is an essential step forward in this therapy. Although early phase studies of CMV-specific ACT have demonstrated substantial improvements in the appearance of MRI scans and patients’ survival, these findings are still preliminary, and research is required to broaden the application of this strategy in clinical settings. Additionally, determining the appropriate target group of patients for this approach based on influential markers in clinical responsiveness and, on the other hand, combining CMV-specific ACT with DC-vaccination, CAR T cell therapy, and immune checkpoint inhibitors are of interest to investigate in future studies to develop an efficacious multi-antigen directed approach with minimal side effects.

## Author Contributions

ADS and AS extracted the data, took the lead in writing the manuscript and designing the figures and tables. HS designed the figures. FM, MG, and NS reviewed and edited the manuscript. OB and BB supervised and helped to develop the research question. All authors contributed to the article and approved the submitted version.

## Conflict of Interest

The authors declare that the research was conducted in the absence of any commercial or financial relationships that could be construed as a potential conflict of interest.

## Publisher’s Note

All claims expressed in this article are solely those of the authors and do not necessarily represent those of their affiliated organizations, or those of the publisher, the editors and the reviewers. Any product that may be evaluated in this article, or claim that may be made by its manufacturer, is not guaranteed or endorsed by the publisher.
